# Change of Health and Change of Preferences on Life-Sustaining Treatment: Evidence From a Longitudinal Study

**DOI:** 10.1093/geroni/igab046.069

**Published:** 2021-12-17

**Authors:** Yifan Lou, Mercedes Bern-Klug, Jinyu Liu

**Affiliations:** 1 Columbia University, New York, New York, United States; 2 University of Iowa, Iowa City, Iowa, United States; 3 Columbia University, Columbia University, New York, United States

## Abstract

Background: Decision-making for end-of-life (EoL) care is not a one-off choice. Older adults may change their preferences for life-sustaining treatments along their health continuum. Guided by prospect theory, we hypothesize that perceived change in health status is a driver behind preference changes. Method: Health and Retirement Study Wave 2012 to 2018 data. Sample is limited to 5,646 older adults who reported whether they requested to limit treatment in living will during two waves of data. Two possible preference changes were tested: from limited to default care and from default to limited care. Change in health status was indicated by changes (1=same, 2=improve, 3=decline) in physical pain, general health, activities of daily living, instrumental activities of daily living (IADL), and number of diagnoses. Multilevel logistic regression models were used to understand how change of health status was related to changes in EoL preferences. Results: 700 older adults changed their preferences some time in 8 years. Those who changed their preferences are more likely to be older and not married, and to have lower socioeconomic background. Older adults who experienced deteriorated pain levels were more likely to change their preferences from default to limited care (OR=3.77, p<.05) and less likely to change from limited to default care (OR=0.63, p<.05). Change in IADL is also a significant predictor of change of preferences. Implication: The findings highlight the importance of periodic reassessment of EoL care preferences with older adults. We discuss policy and practice implications regarding health changes as underlying mechanisms of preference changes.

